# Isolation, characterization and comparative genomics of potentially probiotic *Lactiplantibacillus plantarum* strains from Indian foods

**DOI:** 10.1038/s41598-022-05850-3

**Published:** 2022-02-04

**Authors:** Sarvesh Surve, Dasharath B. Shinde, Ram Kulkarni

**Affiliations:** 1 Symbiosis School of Biological Sciences, Symbiosis International (Deemed University), Lavale, Pune, 412115 India; 2 Present Address: Molecular and Cellular Biology Program, Dartmouth College, Hanover, NH 03755 USA

**Keywords:** Bacterial genomics, Applied microbiology, Bacteria, Bacteriology, Cellular microbiology, Environmental microbiology, Industrial microbiology

## Abstract

*Lactiplantibacillus plantarum* is one of the most diverse species of lactic acid bacteria found in various habitats. The aim of this work was to perform preliminary phenotypic and genomic characterization of two novel and potentially probiotic *L. plantarum* strains isolated from Indian foods, viz., dhokla batter and jaggery. Both the strains were bile and acid tolerant, utilized various sugars, adhered to intestinal epithelial cells, produced exopolysaccharides and folate, were susceptible for tetracycline, erythromycin, and chloramphenicol, did not cause hemolysis, and exhibited antimicrobial and plant phenolics metabolizing activities. The genetic determinants of bile tolerance, cell-adhesion, bacteriocins production, riboflavin and folate biosynthesis, plant polyphenols utilization, and exopolysaccharide production were found in both the strains. One of the strains contained a large number of unique genes while the other had a simultaneous presence of glucansucrase and fructansucrase genes which is a rare trait in *L. plantarum.* Comparative genome analysis of 149 *L. plantarum* strains highlighted high variation in the cell-adhesion and sugar metabolism genes while the genomic regions for some other properties were relatively conserved. This work highlights the unique properties of our strains along with the probiotic and technically important genomic features of a large number of *L. plantarum* strains.

## Introduction

Lactic acid bacteria (LAB) are one of the most important groups of food-related bacteria that are widely used in the food, probiotic, dairy, and beverage industries. These applications are due to generally recognized as safe (GRAS) status of LAB as well as the peculiar properties of these bacteria that make them useful for such applications. In terms of probiotic properties, certain strains of lactobacilli are used for the applications such as treating gastric diseases, immune modulation, and prevention of colonization of harmful bacteria^1,2^. Certain traits that such potentially probiotic lactobacilli display include the ability to survive in low pH and bile, higher hydrophobicity, adhesion to the colon epithelium, production of conjugated linoleic acid, production of exopolysaccharides, etc^3–5^. *Lactiplantibacillus plantarum*, one of the members of lactobacilli, is found in various environments and harbors a variety of probiotic properties. This homofermentative species is known for its highly variable strains possessing diverse phenotypes and variable genomes. There are many incidences of its isolation from various foods including plants, meat, and fermented products^6^. They are normal inhabitants of the gut microsystem and have been isolated from the guts of various mammals, fruit flies, and honeybees. The vaginal microsystem has also shown the presence of *L. plantarum* and they have been evaluated for their probiotic properties^7^. A few strains of *L. plantarum* such as 299v which was isolated from human intestinal mucosa and Lp01 are commercially used as probiotics. The genome size of *L. plantarum* strains is in the range of 3–3.6 million base pairs which is higher compared to other LAB^8^. In a comparative genomic study establishing a connection between evolution and habitat, *L. plantarum* has been identified to be part of the nomadic lifestyle^9^.

*Lactiplantibacillus plantarum* has been frequently found in Indian fermented foods such as idli and dosa batter, and sorghum-based fermented products^10–12^. It has also been reported from fermented vegetable products such as gundruk, sinki, khalpi, inziangsang and xaj-pitha from North-East India^13,14^. In contrast to these reports and the rich diversity of the fermented foods in India, not many studies have been carried out on the genome sequencing and analysis of *L. plantarum* from India*.* This is also reflected in the fact that of the total 593 *L. plantarum* genomes available on the PATRIC database, only 16 are reported from India. Recently, genomic characterization of *L. plantarum* isolated from dahi and kinema revealed their putative bacteriocin production and probiotic potential^15^. Additionally, Indian *L. plantarum* isolates Lp91from human gut and JDARSH from sheep milk have also been sequenced for their genome^16,17^. In this paper, we describe the isolation, phenotypic and genotypic characterization, and comparative genomics of two diverse *L. plantarum* strains isolated from different food sources in India. These isolates were also shown to have high probiotic potential in-silico as well as in-vitro. Additionally, we report variation in *L. plantarum* strains with respect to the presence of genomic determinants of cell adhesion, carbohydrate metabolism, vitamin biosynthesis, and metabolism of plant phenolics by mining the publicly available whole-genome sequence data of 147 strains.

## Materials and methods

### Samples, bacterial cultures, and growth conditions

The bacterial cultures were isolated from the batter used for making dhokla (Indian fermented food) and a jaggery sample from the Indian states of Gujarat and Maharashtra, respectively. The food samples were inoculated in mMRS broth (deMan, Rogossa, Sharpe media supplemented with 0.05% L-cysteine) and streaked on mMRS agar after 24 h of incubation at 37 °C. The isolates identified as *Lactiplantibacillus plantarum* by 16S rRNA gene sequencing were selected for further characterization. The strains were regularly grown in mMRS medium and grown at 37 °C. *Escherichia coli* MTCC730, *Pseudomonas aeruginosa* ATCC27853, *Enterococcus faecalis* ATCC14506, and *Listeria monocytogens* ATCC19115 were grown in nutrient broth.

### Whole-genome sequencing

Genomic DNA was isolated using Wizard Genomic DNA Purification Kit (Promega Inc, USA) following the manufacturer’s protocol. Whole-genome sequencing using Illumina Nextseq500 platform to generate paired-end 150 bp sequences was outsourced. The raw reads were assembled using Unicycler^18^ and the analysis and visualization of the genomes were performed using genome analysis tools on PATRIC BRC^19^ (https://patricbrc.org/). Plasmid contigs were identified using Platon tool^20^. *Lactiplantibacillus plantarum* WCFS1 was used as a reference to sequentially order the whole genome contigs using Mauve Contig Mover^21^. The contigs were subjected to ORF prediction and annotation using PATRIC BRC^22^.

### Phenotypic characterization

The isolates were analyzed for their ability to thrive separately in mMRS having 0.3% oxgall (Merck) and mMRS with pH adjusted to 3.9 using hydrochloric acid by CFU/ml counts after three-hour exposure. The ability of the strains to utilize various sugars was assessed by growing them in minimal mMRS medium containing 2% (w/v) sugars viz., lactose, maltose, arabinose, melezitose, melibiose, raffinose, salicin, sorbitol, trehalose, sucrose, mannose, and fructose and comparing OD_600_ with those obtained with 2% (w/v) glucose. Nitrate reduction was examined on nitrate broth using sulphanilic acid and α-naphthylamine with zinc dust. H_2_S production, arginine dihydrolase activity, and urease activity were analyzed on TSI agar, arginine dihydrolase broth, and urea agar, respectively. Catalase activity was assessed using 15% H_2_O_2_ on a glass slide. The pH reduction was examined after 3 days of the growth in 10% skim milk media at 37 °C.

### Technological characterization

Cell adhesion assay was performed by seeding 10^7^ CFU of the bacteria on 21-days post confluent human colorectal adenocarcinoma cells (HT-29). Bacteria were allowed to adhere to HT-29 cells for 1 h at 37 °C. Non-adhered bacterial cells were removed by washing with PBS, adhered bacteria were released by trypsinization, and the bacterial cell count was estimated by determining CFU^23^. Antimicrobial activity was assessed using the agar spot diffusion method by spotting the supernatants of the overnight grown cultures on nutrient agar plates and pouring pathogens mixed in soft nutrient agar over it^24^. *Escherichia coli* MTCC 728, *L. monocytogenes* ATCC 19115, *E. faecalis* ATCC 14506, *P. aeruginosa* ATCC 27853 were used as the test pathogens and the zone of inhibition was considered as the measure of antimicrobial activity.

Exopolysaccharide (EPS) production of both the strains was determined as described earlier with some modifications^25^. Briefly, the strains were grown in 50 ml skim milk (100 g/l) supplemented with sucrose (50 g/l), casitose (10 g/l) and K_2_HPO_4_ (1 g/l) for 72 h. The coagulants were broken and pH was adjusted to 7.5 with 2 M NaOH followed by the addition of pronase (0.1 g/l) and thiomersal (1 g/l) and incubation at 37 °C for 24 h. EPS were precipitated from the supernatant by adding three volumes of cold absolute ethanol and incubating at 4 °C for 24 h. The precipitates were air-dried and dissolved in deionized water. The crude ESP solutions were further treated with 12% trichloroacetic acid stored at 4 °C for 24 h. The proteins were pelleted by centrifugation and the supernatant were subjected to dialysis for 3 days at 4 °C against deionized water. Dialysed EPS fractions were again precipitated with three volumes of cold absolute ethanol. EPS yields were determined by phenol–sulphuric acid method in a 96-well microplate^26^. The monosaccharide composition was determined using gas chromatography after converting acid hydrolyzed EPS to its alditol acetate derivatives^27^.

Folic acid production by the strains was analysed using reporter strain *Lacticaseibacillus rhamnosus* DSM 20021 which needs folic acid for growth^28^. DKL3 and JGR2 were grown in folic acid casei medium (Himedia Laboratories) and cell free supernatants (CFS) were made. Growth of *L. rhamnosus* DSM 20021 in folic acid casei medium supplemented with 20% CFS was assessed and considered as the indicator of folic acid production. Feruloyl esterase activity was assessed using mMRS agar plates containing ethyl ferulate (1% w/v in dimethyl formamide) and observing the zone of clearance. The mMRS broth was supplemented with 4 mg/ml tannic acid (TA) for detecting tannase activity and 4 mg/ml gallic acid (GA) for detecting gallate decarboxylase activity, separately and the cultures were inoculated for 24 h. The culture supernatants of tannase activity assay were analysed by TLC with chloroform–ethyl acetate-acetic acid (50:50:1) as the mobile phase and 1% ferric chloride in methanol as the developing reagent^29^. The culture supernatants of gallate decarboxylase activity assay were extracted using ethyl acetate followed by evaporation of solvent and the products were assessed by GC–MS (Agilent, 8890 GC System coupled with 5977B MSD) after TMS-derivatization using BSTFA + 1%TMCS (N,O-Bis(trimethylsilyl)trifluoroacetamide).

The unique GH68 (FTF) gene from DKL3 was amplified from genomic DNA, cloned in EcoRI site of pET30b vector and expressed in *E. coli* BL21 (DE3). The protein purified by Ni-NTA affinity chromatography was assayed by monitoring glucose release from sucrose by DNSA (3,5-Dinitrosalicylic acid) method.

### Safety assessment

The hemolytic activity of the isolates was assessed by streaking them on anaerobic blood agar plates (Himedia Laboratories). The antibiotic sensitivity of the isolates was analyzed using Ezy-MIC strips (Himedia Laboratories) carrying tetracycline, gentamycin, vancomycin, clindamycin, and trimethoprim. The MIC values were compared with the cut-off values defined by EFSA^30^. Antibiotic resistance (AR) genes were annotated using comprehensive antibiotic resistance database (CARD) analysis^31^. The proteins involved in the biogenic amine production (Table S1) were also annotated using BLASTp with E-value threshold of 1E-10.

### Genome characterization

The insertion elements were identified by subjecting the ordered contigs to ISfinder using BLASTn v2.2.31 with an E-value threshold of 1e−50^32^. The Antibacterial Biocide and Metal Resistance Genes Database (BacMet) v2.0^33^ was used to predict the biocide and metal resistance genes using BLASTx v2.2.26 against the experimentally characterized (n = 753) and predicted (n = 155,512) resistance gene with an E-value threshold of 1e−100. CRISPR-Cas elements were determined by the CRISPERCasFinder^34^. Identification of prophage loci was carried out using PHASTER^35^.

The presence of the genes encoding bile salt hydrolase (Bsh) and esterases involved in the hydrolysis of phenolics in DKL3 and JGR2 genomes was assessed using BLASTp tool with the amino acid sequences of the previously characterized genes as the queries and an E-value threshold of 1E-10 (Table S1). Proteins involved in vitamin biosynthesis mentioned in previous reports^36^ were similarly identified. EPS clusters were identified as described earlier^37^. BAGEL4 was used to identify the bacteriocin-related gene clusters in the genomes of DKL3 and JGR2^38^.

### Comparative genomics

Genome sequences of the *L. plantarum* strains having whole-genome status as complete on NCBI were downloaded (n = 147) and used for the comparative analysis (Table S2). Seven representative strains of *L. plantarum* viz. JDM1, ST-III, WCFS1, ZJ316, P8, 16, and DSM20174 along with DKL3 and JGR2 were used for the whole-genome phylogeny. Protein sequences of these nine strains were compared considering WCFS1 as the reference using the proteome comparison tool available on PATRIC^39^. The genomes of these nine strains were also compared by assessing the presence of PATRIC cross-genus families (PGFam) for the identification of the unique proteins.

All the complete *L. plantarum* genomes along with DKL3 and JGR2 were subjected to BLAST with 15 cell adhesion-related protein sequences^40^ (Table S3) using BLASTp with an E-value, % query coverage, and % identity thresholds of 1E-10, 70%, and 50%, respectively. These genomes were also annotated using dbCAN2.0 for the identification of the carbohydrate-active enzymes as described on CAZy^41,42^. The presence of genes involved in lactose utilization, vitamin biosynthesis and plant phenolics utilization were also assessed across all the 149 strains by BLASTp.

## Results and discussion

Research on the probiotic or food-industry potential of lactic acid bacteria (LAB) has been on the rise in the last couple of decades. In this quest, the genus *Lactobacillus* (basonym) became popular because of the diverse properties and the generally recognized as safe (GRAS) status of the individual species. Recently, lactobacilli have been reclassified in 25 genera to address the diversity among the species and *L. plantarum*, one of the most studied species has been classified under the new genus *Lactiplantibacillus* to further emphasize its association with the plants^43^. This study addresses preliminary characterization of two novel and potentially probiotic *L. plantarum* strains isolated from Indian foods.

### Isolation, identification of bacteria and whole-genome sequencing

In this study, the *L. plantarum* strains were isolated from dhokla batter and jaggery. Dhokla batter is made up of fermented chickpea flour, rice, and ground urad dal whereas jaggery is a form of brown sugar made from sugarcane concentrate. Seven LAB isolates were found in jaggery whereas three isolates were found in dhokla batter. They were identified to be *Lactiplantibacillus plantarum*, *Leuconostoc mesenteroides*, *Enterococcus faecium* by 16S rRNA gene sequencing (data not shown). Two of these *L. plantarum* isolates named DKL3 (from dhokla) and JGR2 (from jaggery) were selected for further characterization. The whole genomes of DLK3 and JGR2 were sequenced with a coverage greater than 200-fold. The genome sizes of DKL3 and JGR2 were 3,283,055 and 3,131,275 bp, respectively, with the GC content of about 44% (Table 1 and Fig. S1). Both the genome sizes and GC content fell in the range of 3–3.6 Mb and 44–45% respectively, that have been reported for this species ^9^. To the best of our knowledge, this is the first report of *L. plantarum* isolation from these food sources. Previously, *L. fermentum* was isolated from dhokla batter and exopolysaccharide production of those isolates was evaluated^44^.

**Table 1:** General statistics of whole-genome sequencing of *L. plantarum* DKL3 and JGR2.

Isolate	No. of contigs	Raw read fold coverage	N50	L50	Genome Size (bp)	CDS	rRNA operon	tRNA genes	GC%	OrthoANI
*L. plantarum* DKL3	110	450	236,088	6	3,283,055	3258	2	58	44.33	98.88
*L. plantarum* JGR2	106	238	86,465	13	3,131,275	3094	2	58	44.53	

### Genome characterization and plasmid analysis

The IS elements in both the genomes were detected by ISfinder. DKL3 and JGR2 showed the presence of 12 and 10 IS elements, respectively, of which 5 and 7, respectively, were complete (Table S4). Both strains contained IS1165 (family ISL3) and ISLpl1, ISPp1 (family IS30). DKL3 had three extra members belonging to IS30 and IS3 family elements; whereas; JGR2 had one extra member belonging to ISL3. IS elements are known to play a role in creating genetic diversity aiding in the adaptation of the microbes^45^. One putative P-type ATPase gene involved in copper translocation across the membrane was found in both strains using the BacMet database (data not shown). When mined for CRISPR-Cas elements, only DKL3 showed the presence of three CRISPR elements, and no Cas cluster was found in any of the genomes. Two intact prophage regions were identified in JGR2, whereas one complete and two incomplete regions were found in DKL3 (Table S5).

Upon the analysis through the Platon tool, 33 plasmid contigs harbouring 246 CDS were found in DKL3 whereas 10 contigs having 102 CDS were found in JGR2 (Table S6). More than 70% of these CDS could be annotated via eggNOG database reflecting ‘replication, recombination and repair’ and ‘function unknown’ as the top-most categories contributing to more than 40% of the CDS to the annotated plasmidome. Some of the notable plasmid-encoded proteins were glycine betaine transporter, glucansucrase, fructansucrase, major facilitator transporters, metal ion uptake proteins, and toxin-antitoxin systems. Previously, we reported the presence of some such genes at a higher frequency on the plasmids of *L. plantarum* than any other species^46^. Overall, these results indicate the contribution of plasmids to the stress resistance as well as potentially technical properties of DKL3 and JGR2.

### Comparative genomics

**Figure 1:**
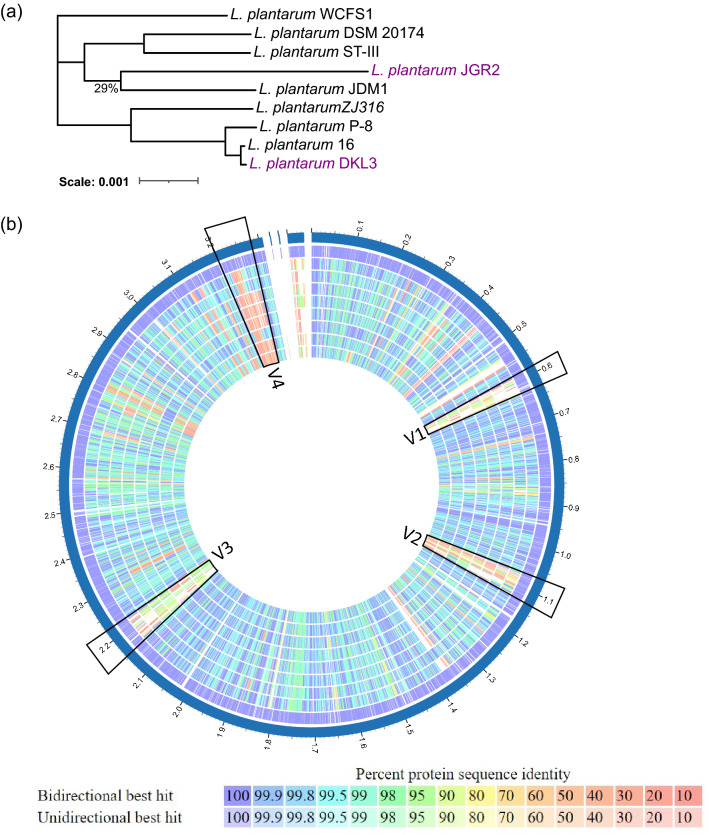
Genome and proteome comparison of *L. plantarum* DKL2 and JGR2 with the reference strains. (**a**) Core genome-based phylogeny performed by neighbour-joining method and illustrated using iTOL (v6, https://itol.embl.de/). Only the bootstrap values < 100% are shown in figure. (**b**) Proteome comparison using PATRIC (v 3.6.12, https://www.patricbrc.org/) showing the genomic regions (V1-V4) encoding the variable proteins. List of tracks, from outside to inside: *L. plantarum* WCFS1, ST-III, JDM1, P-8, 16, ZJ316, DSM 20174, DKL3, and JGR2.

The genomes of DKL3 and JGR2 along with seven other strains of *L. plantarum* were subjected to core genome-based phylogeny. DKL3 and JGR2 clustered separately from each other suggesting their diverse nature (Fig. 1a) and showed clustering of DKL3 with strain 16 and that of JGR2 with JDM1. The proteomes of these nine strains were also compared with a reference strain *L. plantarum* WCFS1 which is well-characterized to have potentially probiotic properties (Fig. 1b). This analysis resulted in the identification of four regions of high variability. Regions V1 (lp_0624 to lp_0683) and V3 (lp_2398 to lp_2480) were found to be phage-related proteins, region V2 (lp_1176 to lp_1233) contained exopolysaccharide synthesis-related proteins; whereas, region V4 (lp_3590 to lp_3650) contained sugar metabolism-related proteins. These variable genomic regions are consistent with those previously reported^47^.

Using PATRIC BRC, the unique proteins were identified as the members of unique PGFam across the selected strains (Table S7 and S7, supplementary files). *Lactiplantibacillus plantarum* WCFS1 which is a well-characterized and potentially probiotic strain had the highest number (32) of unique proteins followed by JGR2 (23), and DKL3 (7). Most of the unique proteins found in DKL3 and JGR2 were phage and sugar metabolism-related, which are known to be variable in *L. plantarum*^47^. This might result in the sugar metabolism of our strains being different from the seven other well-characterized strains including some of the established probiotic candidates (Tables S7 and S8, supplementary files). Since carbohydrate metabolism is strongly associated with the prebiotic, probiotic, and industrial properties through the exopolysaccharides production and utilization oligosaccharides^1,8^, the potentially unique features of JGR2 and DKL3 in this aspect are being investigated. Pyruvate dehydrogenase (quinone) and heme-transporter (IsdDEF) were also found to be two of the unique proteins in our isolates amongst the selected strains. Heme-transporter IsdDEF is an ABC transporter for heme transport across the membrane and has been well characterized in *S. aureus*^48^. Both these genes are likely to contribute to the respiratory growth with enhanced biomass and higher yields of value-added chemicals including diacetyl and acetoin^49^. Thus, it will be worth analysing the plausible uniqueness of JGR2 in this regard in comparison to the established probiotic and industrial candidates not having these genes.

### Phenotypes, technological properties and their genetic determinants

The isolates did not show nitrate reduction, H_2_S production, arginine dihydrolase activity, urease activity, and catalase activity. The DKL3 cell biomass was yellowish and sticky and the colonies did not disperse easily in the liquid medium whereas JGR2 pellet was white and dispersed relatively easily (Fig. S2). The color variation displayed by both strains is likely due to variable carotenoid production. A previous study showed the involvement of crtNM operon in the carotenoid synthesis which rendered yellow color to the *L. plantarum* isolates^50^. Even though our isolates showed color variation, the crtNM operon was present in both the isolates (data not shown).

#### Sugar utilization

**Figure 2:**
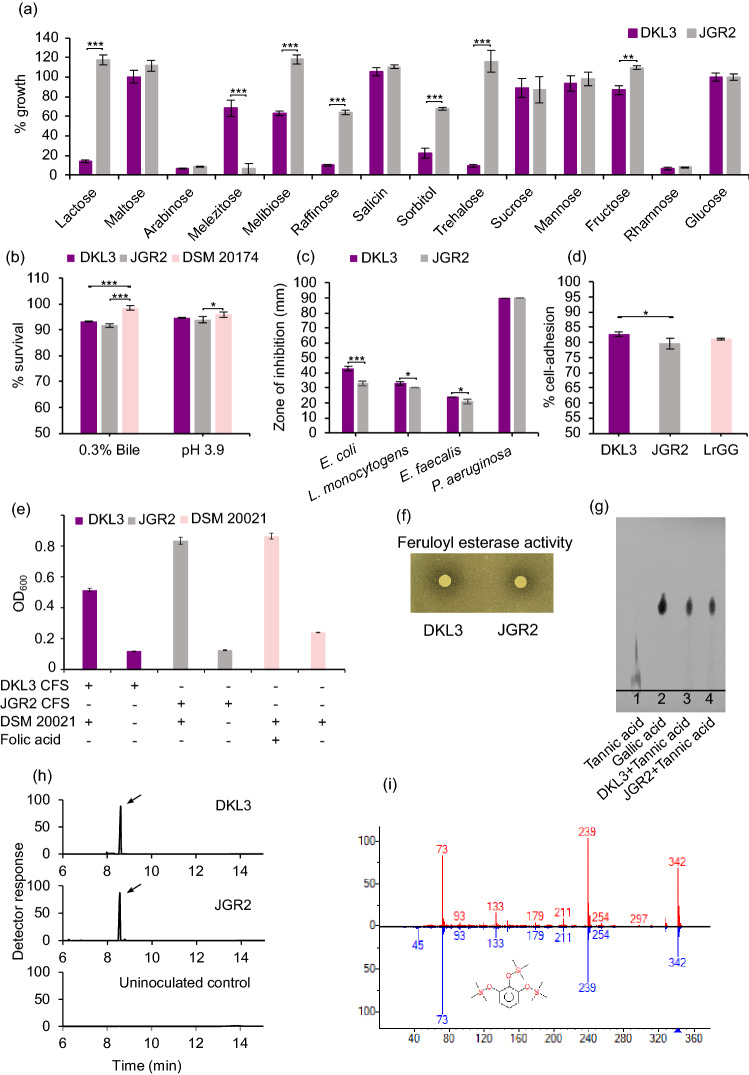
Phenotypic characterization of *L. plantarum* DKL3 and JGR2. (**a**) Sugar utilization. Values denoted are percentage as compared to glucose. (**b**) Bile and low pH survival. (**c**) Antimicrobial activity against various pathogens. (**d**) Adhesion to the HT-29 cell line. (**e**) Folate production as assessed by the growth of *L. rhamnosus* DSM 20021 in folic acid deficient medium supplemented with cell-free supernatant (CFS) of DKL3 and JGR2. (**f**) Feruloyl esterase activity as seen as the zone of clearance surrounding the growth of the strains in mMRS agar containing ethyl ferulate (**g**) Tannase activity as assessed by TLC of the products formed upon incubating the strains with tannic acid. (**h**) Gallate decarboxylase activity as assessed by GC–MS analysis of the TMS-derivatized reaction products formed upon incubating the strains with gallic acid. 3TMS-pyrogallol peak is indicated by the arrows in the total ion chromatograms. The mass spectrum of the 3TMS-pyrogallol peak in (**h**) is indicated in top-panel of (**i**) whereas the bottom panel represents 3TMS-pyrogallol spectrum in the mass spectral library. All values are mean of triplicates and error bars denote standard deviation. Asterisks denote the values which are significantly different from each other as determined by unpaired Student’s t-test for a and c and two-way ANOVA for (**b**) and (**d**) (*p* ≤ 0.05, *; *p* ≤ 0.01, **; *p* ≤ 0.001, ***).

Both the strains were able to utilize maltose, salicin, sucrose, mannose, and fructose to a similar extent as glucose but neither of them was able to utilize arabinose and rhamnose (Fig. 2a). Melezitose was only utilized by DKL3; whereas, lactose, raffinose, sorbitol, and trehalose were exclusively utilized by *L. plantarum* JGR2. In general, high variability in the utilization of some sugars is reported in *L. plantarum* strains. The common and differential pattern observed for DKL3 and JGR2 was in accordance with such differences reported, for example, in the case of *L. plantarum* 299v and ATCC 14917^51,52^. For melezitose, raffinose, sorbitol, and trehalose utilization, the gene cassettes were found in both the genomes despite variable utilization. It has been shown that *L. plantarum* carbohydrate utilization operons are highly variable and are altered depending on the niche^53^. The correlation of these variable sugar utilization with the genome was only possible in the case of lactose, where a lactose utilization gene cassette was found only in JGR2.

The lac region in WCFS1 is identified to be lp_3468 to lp_3470 and encodes for LacS (lactose and galactose permease), LacA (β-galactosidases), LacR (lactose transport regulator)^54^. JGR2 genome consisted of four β-galactosidases, of which one belongs to the GH42 family (*lacA*), and a *lacS*; but lacked *lacR*. The absence of *lacR* might make the lactose utilization in JGR2 constitutive as observed in *L. delbrueckii*^55^. In the case of DKL3, all these genes were absent, justifying its inability to utilize lactose. This operon has also been shown to be involved in utilization of galacto-oligosaccharides (GOS) but the absence of *lacR* has been associated with non-GOS utilization phenotype^56^. Thus, none of our strains might be able to utilize GOS.

**Figure 3:**
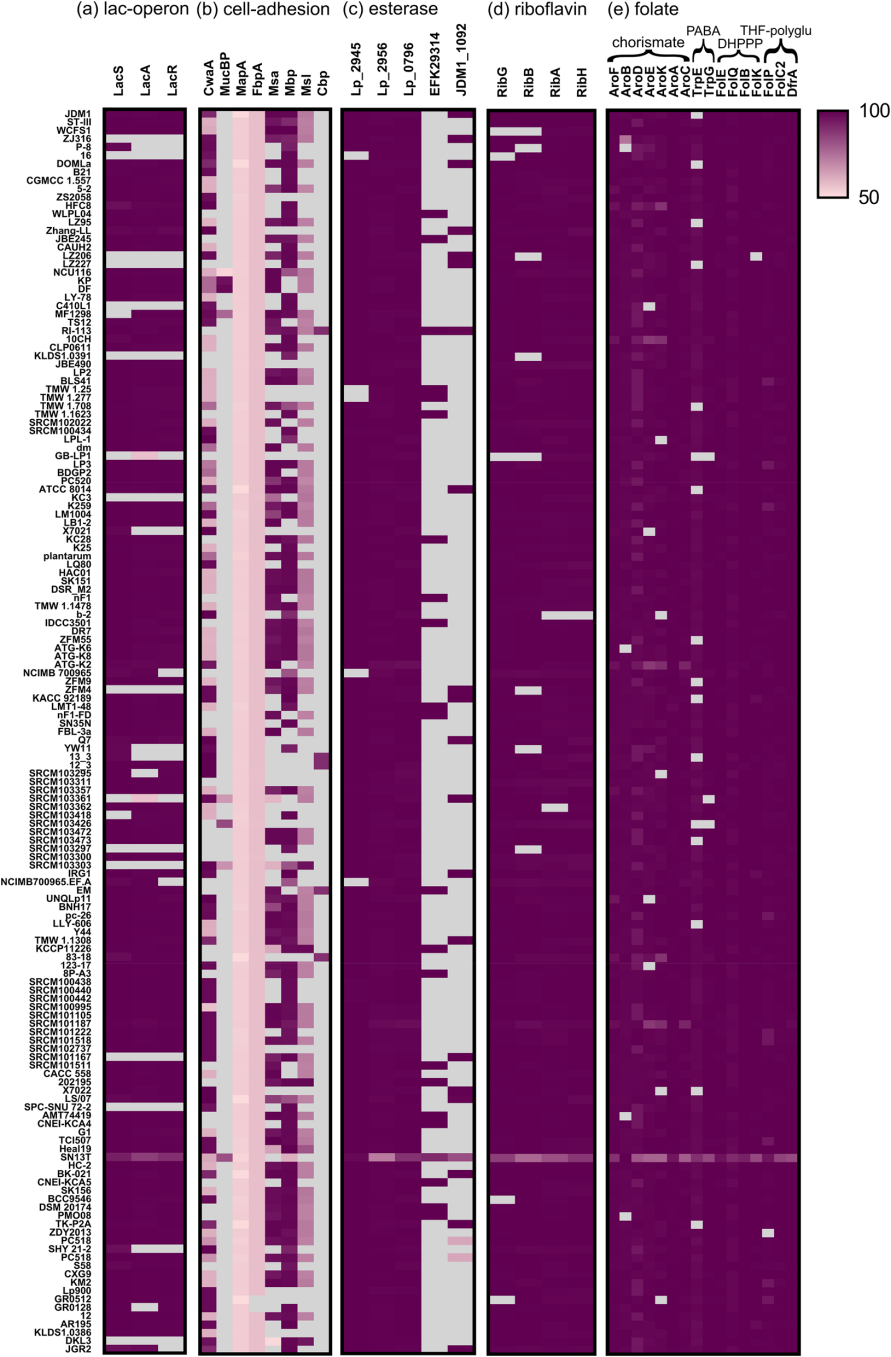
Comparative genomics of *L. plantarum* strains for the presence of genes encoding proteins related to lactose utilization, adhesion, metabolism of phenolics, and vitamin biosynthesis. BLASTp results depicted as heatmaps with grey color denoting no hit and pink to dark purple gradation denoting presence of genes with 50%-100% identity with the query sequences. (**a**) Lac operon, LacS: lactose and galactose permease, LacA: β-galactosidase, LacR: lactose transport regulator. (**b**) Cell-adhesion related proteins, CwaA (cell wall-anchored adhesion-associated protein), MucBP (mucin-binding protein), MapA (mucus adhesion-promoting protein), FbpA (fibronectin-binding protein A), Msa (mannose-specific adhesin), Mub (mucus-binding protein precursor), Msl (mannose-specific lectin), and Cbp (collagen-binding protein). See Table S3 for the details of the proteins used as the query sequences. (**c**) Esterases characterized from *L. plantarum*, Lp_2945 (Gallate decarboxylase from WCFS1), Lp_2956 (Tannase from WCFS1, Lp_0796 (Esterase from WCFS1), EFK29314 (Tannase from ATCC14917) and JDM1_1092 (Esterase from JDM1). (**d**) Riboflavin and (**e**) folate biosynthesis proteins, adapted from^36^.

Since we found correlation between lactose utilization and the presence of the lac operon in JGR2, both of which were not observed in DKL3, we further expanded the assessment of lac operon to 147 strains of *L. plantarum* for which the complete genome sequences were available on NCBI database. In total, 122 (~ 82%) strains were found to have the complete lactose utilization cassette harbouring *lacS*, *lacA*, and *lacR* (Fig. 3a). Including DKL3, only 17strains lacked *lacS*; whereas, 20 strains lacked the *lacA*, as a result these strains might not be able to utilize lactose. Total 23 strains lacked *lacR* out of which three strains contained the other two genes (*lacS* and *lacA*) hence might be able to utilize lactose but not GOS, similar to JGR2.

#### Bile and acid tolerance

Both DKL3 and JGR2 showed < 1 log_10_ CFU reduction after three-hour exposure to 0.3% oxgall and pH of 3.9, separately. This reduction was slightly higher than that observed for *L. plantarum* DSM 20174 under similar conditions (Fig. 2b) but lower than that reported for a commercial probiotic strain, *L. plantarum* 299v as assessed for the shorter exposure duration^57^. These observations suggest that DKL3 and JGR2 might be able to survive in the human gastrointestinal tract during its probiotic usage.

The bile resistance of DKL3 and JGR2 correlates well with the presence of the four genes encoding bile salt hydrolase (Bsh) proteins in their genome that have been characterized from the two highly studied probiotic strains, *L. plantarum* WCFS1^58^ and *L. plantarum* ST-III^59^. None of the genes encoding Bsh from other lactobacilli^60^ or human gut microbiota^61^ were found in DKL3 and JGR2. As *L. plantarum* produces acid in its own environment, one of the contributors to the acid tolerance is F-ATPase which regulates the intracellular pH^62^. Also, Cfa1 (cyclopropane-fatty-acyl-phospholipid synthase), MleS (malolactic enzyme) and HisD (histidinol dehydrogenase) which are also known to play a role in acid resistance of *L. plantarum*^1,63^ were found to be encoded by both the genomes.

#### Antimicrobial activity

DKL3 and JGR2 showed the zones of inhibition against all the test pathogens and the extent of such antimicrobial activity for the given pathogen was similar for DKL3 and JGR2 (Fig. 2c). The highest inhibition was in the case of *P. aeruginosa* ATCC 27853 while the lowest was seen for *E. faecalis* ATCC 14506.

When mined for the presence of genomic determinants of bacteriocin production, both the strains were found to have plantaricin E/F genes (Fig. S3). Additionally, JGR2 also showed the presence of two lactococcin (ComC, member of class IIc), plantaricin A (member of class IId) and J (member of class IIB) genes (Fig. S3). Both plantaricin E/F and plantaricin J/K are class IIb two-peptide bacteriocins where both the peptides act synergistically to confer antimicrobial activity^64,65^. Since the gene for plantaricin K was not found, plantaricin J might not be active. Along with the structural genes, the plantaricin E/F loci in both the genomes contained genes encoding ABC transporters (LanT and HlyD), a two or three-component system (HPK, plnC (only seen in JGR2), plnD), immunity protein (plnI), biosynthesis proteins (plnS and/or plnY), and DNA helicase IV. The sequence of these genes in DKL3 was similar to JDM1; whereas, that in JGR2 was similar to WCFS1. Since the bacteriocin clusters/operons in DKL3 and JGR2 appear to be complete, both the strains are possibly able to synthesize, process and secret the plantaricin E/F and this property could contribute to the antimicrobial activity showed by the isolates.

Both the isolates showed acid production with a pH drop till 4.2 for DKL3 and 4.6 for JGR2 in 10% skim milk having the initial pH of 6.5. Thus, in addition to the bacteriocin production, the antimicrobial activity displayed by the isolates can also be attributed to the production of organic acids. Antimicrobial properties are beneficial in food preservation during fermentation and also as an important probiotic characteristic^66^.

#### Adhesion to human intestinal epithelial cells

The extent of adhesion to the HT-29 intestinal epithelial cells, of DKL3 (82.8%), and JGR2 (79.6%), were similar to that of an established probiotic strain *Lacticaseibacillus rhamnosus* GG as determined by us (81.1%) (Fig. 2d) and as reported earlier (80.8%)^67^. This extent was also higher than that reported earlier for another probiotic strain, *L. plantarum* 299v (24%)^57^. This observation suggests the possibility of equivalent or even better ability of DKL3 and JGR2 to colonize the gut in comparison to the established probiotics.

The adhesion properties of the probiotic bacteria are highly important for the gut colonization and are based on the cell surface characteristics of the bacteria. Over the years, various cell adhesion-related proteins have been characterized from lactobacilli and have been thought to be required for colonization in the host^40^. The genomes of DKL3 and JGR2 were searched for the presence of genes encoding such proteins^40^ (Table S3). Both the strains showed the presence of the genes encoding both cell wall-anchored adhesion-associated protein (CwaA) (96% similarity) and mucus-binding protein precursor (Mbp) (99% similarity). DKL2 and JRG2 were also found to encode fibronectin-binding protein A (FbpA) with low identity (< 58%). Furthermore, mannose-specific lectin (Msl) was found to be encoded by the DKL3 genome with 83.8% identity. Based on the earlier studies, all these proteins appear to be associated with the probiotic properties of the lactobacilli. Specifically, recombinantly expressed CwaA in *Lactococcus lactis* has been shown to be involved in adhesion to the colonic epithelial cells as well as in the exclusion of pathogen^47^. Similarly, recombinant expression of two of the six mucous binding domains of *L. plantarum* Mbp in *E. coli* has been shown to exhibit very high adhesion to rat, pig, and human intestinal tissues and also inhibition of pathogen binding^68^. Also, purified FbpA from *L. casei* has been characterized to show adhesion to immobilized fibronectin^69^. In *L. plantarum* CMPG5300, Msl was shown to be required for adhesion to the vaginal epithelial cells and other cell adhesion properties such as auto-aggregation, biofilm formation, and binding to mannosylated glycans^70^. Thus, the in vitro adhesion properties and the presence of the required genes suggests the potential of both the DKL3 and JGR2 to colonize in the human gut. The presence of msl in DKL3 also suggests that it can have the potential of colonizing in the vagina and subsequently exhibiting probiotic effects.

Additionally, we assessed 147 genomes of *L. plantarum* available in the NCBI database for the presence of these adhesion-related genes (Fig. 3b). *Lactiplantibacillus plantarum* strains have previously been shown to have either of the two types of CwaA^47^. In accordance with this observation, the majority of the strains that we analyzed had Group I CwaA (107 strains), a few had Group II CwaA (30 strains); whereas, the remaining few strains did not contain this protein. Similarly, a protein with about 57.6% similarity to FbpA was present in all but two strains. A protein showing about 54% identity with MapA from *Limosilactobacillus reuteri* but annotated as transporter substrate-binding protein was present in all the *L. plantarum* strains analyzed. Proteins similar to mucin-binding protein (MucBP) (8 strains with 52–98% identity), and collagen-binding protein (Cbp) (5 strains with 88–89% identity), were scarcely found across *L. plantarum* strains and were annotated differently. The presence of adhesion-related protein of one or the other type in a large majority of the *L. plantarum* strains suggests that they might be able to colonize in the animal hosts.

#### Safety of isolates

Although *L. plantarum* has a GRAS status, the safety of each stain for usage in human consumption needs to be determined ^71^. To establish food safety, DKL3 and JGR2 were assessed for their antibiotic resistance at the phenotypic and genomic levels, hemolysis activity at phenotypic levels, and the biogenic amine biosynthesis at the genomic levels. Antibiotic MIC test revealed that both the strains were sensitive to tetracycline, chloramphenicol, clindamycin, and trimethoprim and resistant to gentamicin and vancomycin (Table 2). Campedelli et al. (2019) reported phenotypic resistance to tetracycline and chloramphenicol in a majority of *L. plantarum* strains in their panel. This fact suggests the superiority of JGR2 and DKL3 on this parameter. Resistance to gentamicin and vancomycin is considered to be intrinsic and is well-studied in lactobacilli^72^. When mined for AR genes, those conferring resistance to tetracycline, chloramphenicol, and erythromycin were found in both strains in spite of having the phenotypic susceptibility to the former two antibiotics. This observation is also consistent with the earlier report which showed the presence of chlorapmphenicol resistance gene in a majority of the lactobacilli strains which were susceptible to this antibiotic^72^. *Aac(6’)-Ian* (aminoglycoside 6'-N-acetyltransferase) which is responsible for aminoglycosides resistance was found in both the genomes with 33% identity (Table S9). This gene might be responsible for the observed gentamicin resistance in DKL3 and JGR2^73^. Vancomycin resistance in both the isolates might be due to the presence in them of *ddl* gene identical to the F-type sequence (phenylalanine residue in active site) which is generally found in vancomycin-resistant *L. plantarum*^72^. Taken together, our results suggest better susceptibility of DKL3 and JGR2 to the commonly used antibiotics with a negative correlation with the presence of antibiotic resistance in some cases as has been shown previously^72^. This situation demands further investigations to understand if these putative antibiotic resistance genes encode for active proteins or play some other roles.

**Table 2:** Antibiotic minimum inhibitory concentrations and resistance of *L. plantarum* DKL3 and JGR2.

Antibiotic	Concentration range tested^#^	MIC cut-off for resistance^#^	MIC obtained^#^		Phenotype category
			DKL3	JGR2	
Tetracycline	0.01–240	32	0.1	0.1	Sensitive
Gentamicin	0.064–1024	16	64	64	Resistant
Vancomycin	0.001–240	nr	> 240	> 240	Resistant
Clindamycin	0.001–240	4	0.001	0.06	Sensitive
Trimethoprim	0.001–240	nd	0.01	0.01	Sensitive
Chloramphenicol^$^	30	4	nd	nd	Sensitive

Cut-off values are based on the guidelines given by European Food Safety Authority (EFSA), 2012.

^#^Values in mg/l.

^$^Sensitivity was determined by disc-diffusion method and MIC not determined (nr: not required; nd: not defined).

The isolates did not show hemolysis on the blood agar plates. Genes involved in biosynthesis of the biogenic amines, viz., histamine, tyramine, putrescine, cadaverine characterized from *Lactobacillus saerimneri* 30a^74^, *Enterococcus faecalis*^75^, *Levilactobacillus brevis*^76^ and *L. saerimneri* 30a^77^ (Table S1) were absent in both DKL3 and JGR2. Hence, the absence of hemolysis, absence biogenic amine synthesis genes, the absence of phenotypic resistance to the commonly used antibiotics, and the intrinsic nature of the resistance to some antibiotics supported the safety of these isolates to be used in food or probiotic preparations.

### Plant phenolics utilization and vitamin biosynthesis

The genomes of DKL3 and JGR2 were further analyzed for the presence of the genes involved in metabolizing the plant phenolics (Fig. 3c). Since the presence of some such genes is a strain-specific feature, 147 *L. plantarum* genomes in the NCBI database were also analysed. An esterase characterized from the potentially probiotic *L. plantarum* WCFS1 that can hydrolyze the feruloyl esters^78^ was found in DKL3 and JGR2 along with all other strains analyzed; whereas, another esterase found only in a few strains of *L. plantarum* and having a broad range of activities on numerous phenolic esters^79^ was found in JGR2 along with 22 other strains. The presence of this broad-range and uncommon esterase might allow better growth of JGR2 in plant-based media such as fermented vegetables in comparison to the established probiotic strain, WCFS1 which does not contain this gene^79^. Similarly, a tannase from another probiotic strain, *L. plantarum* ATCC 14917^80^ was found in all the strains including DKL3 and JGR2; a gallate decarboxylase from *L. plantarum* WCFS1^81^ was present in both DKL3 and JGR2 along with 142 other strains; while, another novel Tannase^82^ was found only in 22 *L. plantarum* strains was absent in our strains (Table S1). Furthermore, the phenotypic assays confirmed the presence of feruloyl esterase, tannase, and gallate decarboxylase (Fig. 2f–i) enzymatic activities in DKL3 and JGR2. Since such enzymatic activities are associated with lowering of the potentially carcinogenic phytochemicals, improving the colonic health, and adaptation of the probiotic lactobacilli to the gut environment^83,84^, DKL3 and JGR2 are likely to offer health benefits upon consumption. Additionally, they also have the potential to be employed as the starter cultures for the fermentation of vegetables and fruits for enhancing the levels of bioactive phenolics^85^.

DKL3 and JGR2 along with 137 other *L. plantarum* strains showed the presence of complete pathway required for riboflavin biosynthesis (Fig. 3d). Similarly, 118 strains including DKL3 and JGR2 harboured all necessary genes for folate biosynthesis (Fig. 3e). Both the strains were able to produce folic acid since their cell-free supernatants supported the growth of *L. rhamnosus* DSM 20021 which is deficient in folic acid biosynthesis (Fig. 2e). Thus, the presence of complete pathways for riboflavin and folate biosynthesis^36^ and the production of folate further suggests the potential application of DKL3 and JGR2 for producing nutritionally enriched fermented foods and as probiotics.

### CAZy families

**Figure 4:**
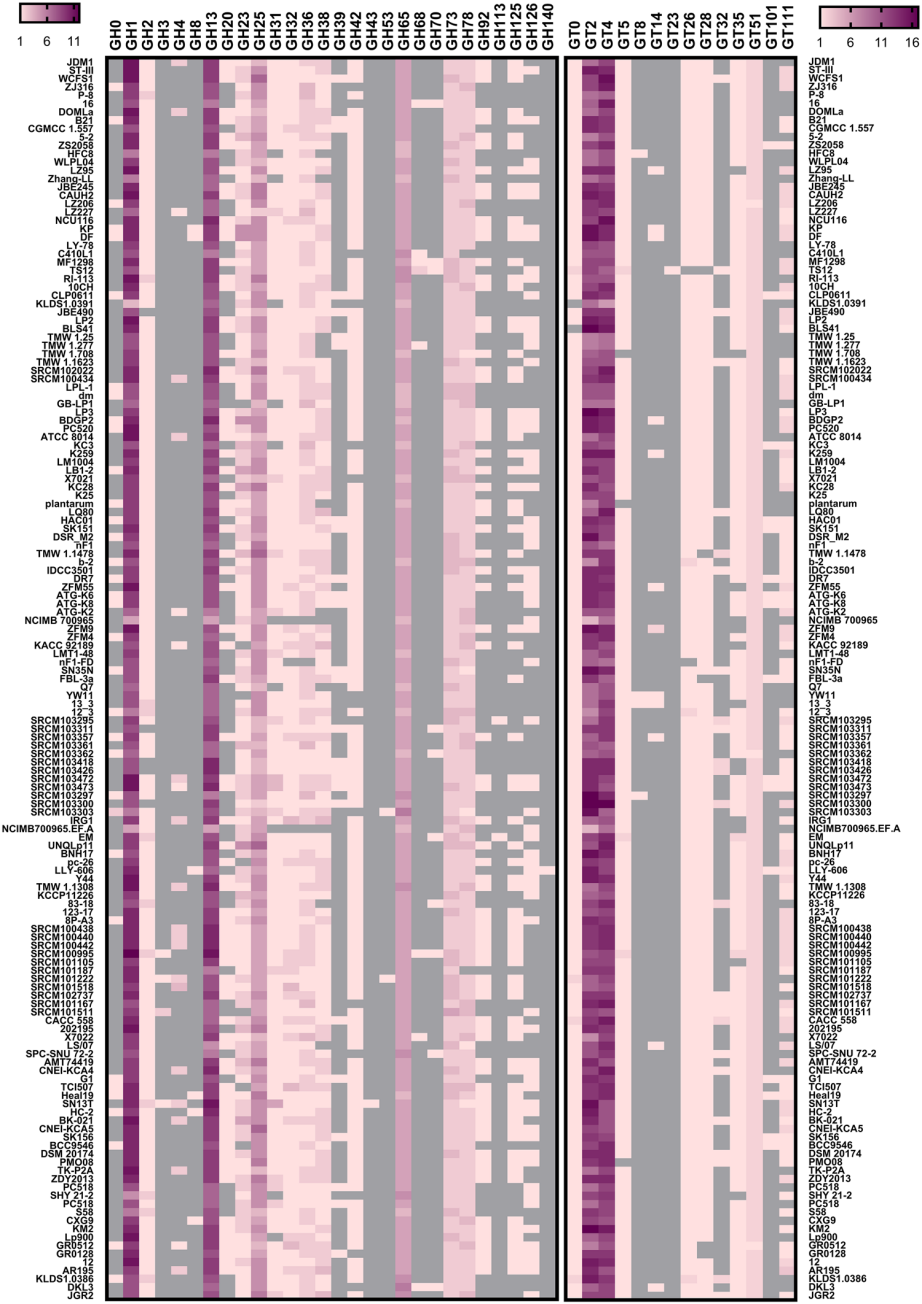
Comparative genomics of *L. plantarum* strains for the presence of CAZy families. dbCAN2 annotation of the glycosyl hydrolase (GH) and glycosyltransferase (GT) families listed in the CAZy database is depicted as a heatmap. Grey color denotes the absence of that family and pink to dark-purple gradation denotes increasing number of members from that family.

Genomes of DKL3 and JGR2 along with those of the 147 *L. plantarum* strains were subjected to annotation by the dbCAN2 server for the identification of carbohydrate-active enzymes (CAZymes). Amongst the glycosyl hydrolases, the families involved in the catabolism of oligosaccharides made up of glucose, galactose, fructose, rhamnose, trehalose, and mannose (GH1, GH2, GH31, GH32, GH36, GH38, GH42, GH65, and GH78), polysaccharides (GH13 and GH32), and cell wall (GH23, GH25, and GH73) were found in more than 90% of the strains (Fig. 4). All these enzymes were also present in JGR2 and DKL3 except for the absence of GH42 in DKL3. Overall, these results underline the ability of DKL3 and JGR2 and well a large number of other strains to utilize various carbohydrates. DKL3 was one of the two strains possessing the highest number (six) of GH65. GH65 enzymes have been characterized to be involved in maltose catabolism in *L. acidophilus* NCFM, *L. sanfranciscensis*, and *L. brevis*^86–88^. Since DKL3 was isolated from the dhokla batter which is likely to be rich in starch, the multiple GH65 hydrolase in this strain might enable it to efficiently use maltose released upon the action of α-amylases which too were abundantly encoded by DKL3 genome. JGR2 was one of about half of the strains having a GH126 member (α-amylase, identified as a unique gene) which was absent in DKL3. Provided that only one GH126 enzyme has so far been characterized and its substrate specificity is still ambiguous^89^, exploring the properties of this enzyme from JGR2 might provide novel insights. DKL3 contained one member each of GH68 and GH70 which were absent in JGR2. These enzymes are involved in homopolysaccharide biosynthesis and were present only in seven and nine of the 147 strains, respectively, highlighting a unique feature of DKL3. Exopolysaccharides which include homo- and heteropolysaccharides are highly important in the technological applications of lactobacilli as well as in their interaction with the host^5^, Thus, assessing the properties conferred by these genes, which were absent in numerous vastly studied *L. plantarum* strains such as WCFS1, JDM1, and ST-III, to DKL3 might reveal novel findings.

Amongst the glycosyltransferases, GT2 and GT4 were the most predominant families and were found in all the *L. plantarum* strains. These genes are mostly associated with the EPS biosynthesis gene clusters in lactobacilli^37^. GT5 and GT35 which were involved in glycogen biosynthesis and degradation, respectively, were present in DKL3 and JGR2 as well as in more than 90% of all the analyzed strains. This observation corroborates with the earlier report on the abundance of these genes in *L. plantarum*^90^, although the biochemical features of these glycogen metabolism enzymes from *L. plantarum* remains to be characterized. GT14 and GT32 were present in 13 and 26 strains, respectively; however, no strain had the simultaneous presence of both these genes. This observation is in agreement with our earlier observation of the mutually exclusive presence of GT14 and GT32 in *Lactobacillus* EPS gene clusters^37^.

### Exopolysaccharide production

Exopolysaccharide (EPS) production is another industrially important trait possessed by LAB. Both DKL3 and JGR2 were able to produce EPS with yields of 397 ± 8.1 mg/l and 461 ± 5.9 mg/l, respectively. The monosaccharide composition was almost identical with glucose (92% and 94%, respectively) as the most abundant monosaccharide followed by galactose (5% and 6%, respectively). Both the strains also had the presence of a very low proportion of mannose. The EPS yields observed for DKL3 and JGR2 fall in the range of 20–600 mg/l reported earlier for lactobacilli and the monosaccharaide composition is also similar to that reported commonly for *L. plantarum*^5^. Both the strains contained an EPS biosynthesis gene cluster highly similar (> 99%) to the *L. plantarum* WCFS1 cluster cps4A-J (data not shown). WCFS1 contains four EPS clusters of which cluster cps4A-J is the most conserved in other *L. plantarum* strains^37,91^. This cluster was previously identified to be involved in contributing to the overall EPS yield and its deletion resulted in less than half yield compared to wild-type^91^. Similar to our earlier observation for other *L. plantarum* strains, DKL3 and JGR2 also did not have *epsA* associated with the EPS gene cluster unlike the host-associated lactobacilli^37^. Both the genomes possessed a gene identical to *lp_1000* from WCFS1 which is involved in biofilm formation^92^ and can act as a putative *epsA*^37^.

**Figure 5:**
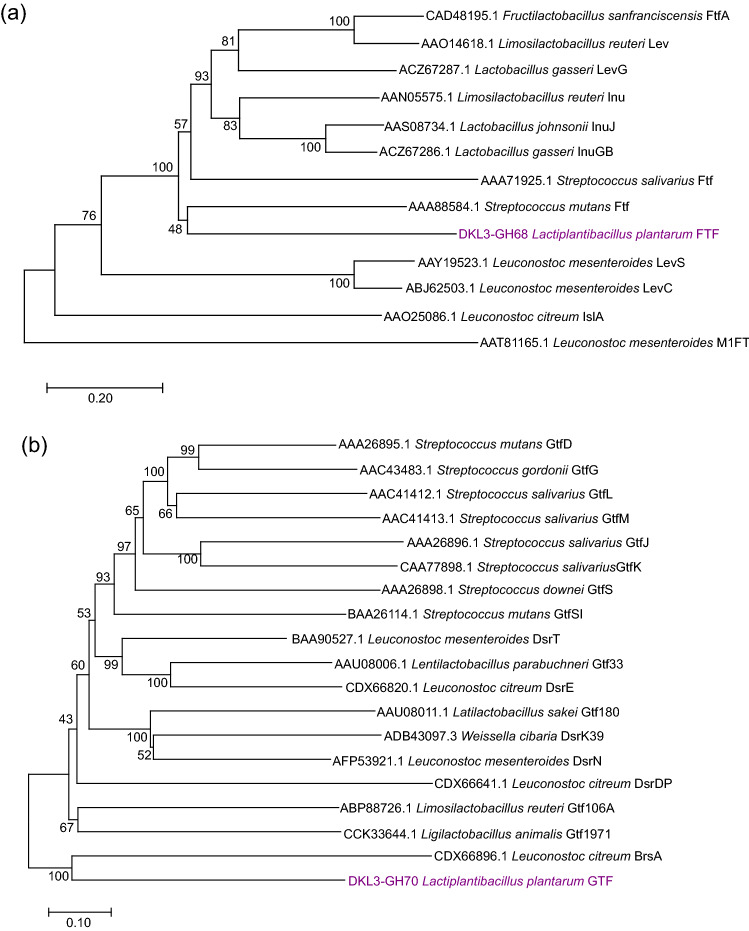
Phylogenetic analysis of fructansucrases (**a**) and glucansucrases (**b**) from *L. plantarum* DKL3 with the respective characterized enzymes from other LAB. The analysis was carried out by neighbour-joining method using MEGA X (https://www.megasoftware.net/)^96^.

**Figure 6:**
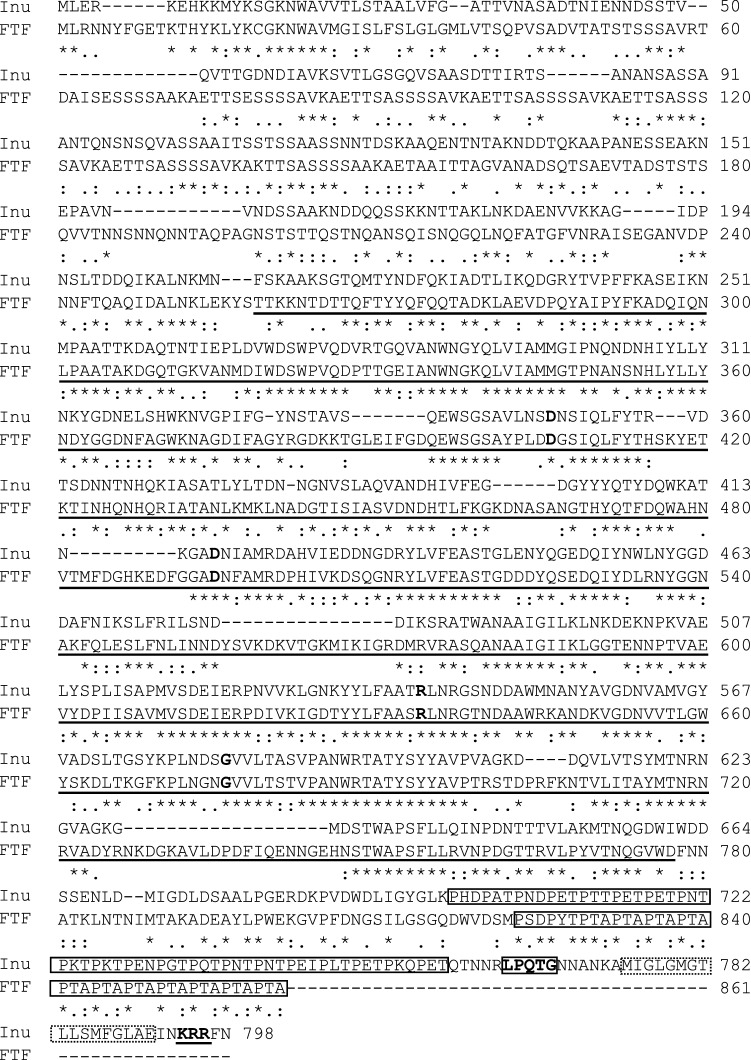
Alignment of fructansucrase (FTF) from *L. plantarum* DKL3 with inulosucrase (Inu) from *L. reuteri* 121 (AAN05575.1)^93^. The domains have been marked according to Van Hijum et al., 2002, the core region of GH68 with a solid line below the sequence (residues 257–777), key amino acids residues (numbers in reference to FTF) D406, D501, R634, and G676 with bold font, and PXX motifs by a box across the sequence (residues 823–861). Additionally, the reference sequence contained the LPXTG motif (denoted by a bold font and thick box across the sequence), a hydrophobic domain (dotted box across the sequence), and positively charged KRR residues (bold and underlined). The alignment was generated using MEGA X (https://www.megasoftware.net/).

We further compared GH68 and GH70 from DKL3 with those characterized from other LAB. The putative GH68 enzyme encoded by DKL3 displayed only 41.7% identity to an inulosucrase characterized from *Limosilactobacillus reuteri* (AAN05575.1)^93^ and much lower identity to the other characterized fructansucrases. On the other hand, the maximum identity showed by GH70 from DKL3 was 78.5% with a dextransucrase characterized from *L. reuteri* (ABQ83597.1)^94^. Phylogenetic analysis of these proteins with those tagged as characterized in the CAZy database revealed clustering of DKL3 fructosyltransferase (FTF) with *Streptococcus mutans* GS-5 fructosyltranferase and DKL3 glucosyltransferase (GTF) with *Leuconostoc citreum* alternansucrase (Fig. 5). Further, the sequence analysis of DKL3 FTF revealed the presence of all the conserved residues found in GH68 enzymes but lack of the cell-wall anchoring motif including the LPXTG and the hydrophobic domain (Fig. 6). This suggests a possibility of extracellular release of this enzyme as has been previously reported for an inulosucrase from *Lactobacillus gasseri*^95^. To assess the activity of DKL3 FTF, the gene was cloned and expressed in *E. coli.* The purified protein showed sucrase activity as assessed by analysing the glucose release from sucrose by DNSA method (data not shown). Considering that glucansucrases have mostly been studied from *Leuconostoc* and no fructansucrase has yet been characterized from *L. plantarum*, further characterization of these GH68 and GH70 enzymes might reveal interesting findings.

## Conclusion

*Lactiplantibacillus plantarum* strains possess larger genomes as compared to the other LAB species. This also translates into the ability of this species to exist in a large number of habitats which is further correlated to the strain-wise variation observed in some of the properties of this bacterium. This scenario demands the detailed analysis of the strains of this species from as many niches as possible to be able to get more insights into the interesting and varying phenotypes that it exhibits. Here we reported isolation and preliminary phenotypic and genomic data of two novel probiotic candidates belonging to *L. plantarum*. To the best of our knowledge, this is the first report on the isolation and genome sequencing of *L. plantarum* from dhokla batter and also on the isolation of LAB and genome sequencing of any bacterium from jaggery. Further studies on functionally characterizing the unique and biotechnologically important genes from these isolates are underway and will shed more light on yet unexplored facets of *L. plantarum.* Our results on comparative genomic analysis of a large number of *L. plantarum* strain suggests the need of establishing genotype–phenotype correlations for a wider array of properties to be able to biologically understand and biotechnologically utilize this fascinating bacterium.

## Supplementary Information

Supplementary Tables.

Supplementary Figure S1.

Supplementary Figure S2.

Supplementary Figure S3.

## Data Availability

The raw NGS reads generated in this project were deposited to NCBI under Bioproject accession PRJNA749646.
