# Supplementary material for: Isolation, characterization and comparative genomics of potentially probiotic Lactiplantibacillus plantarum strains from Indian foods
Source: Sci Rep. 2022 Feb 4;12:1940. doi: 10.1038/s41598-022-05850-3 (PMC8816928; doi:10.1038/s41598-022-05850-3)
Supplement: Supplementary file 2 — Supplementary Figure S1. [file 41598_2022_5850_MOESM2_ESM.pdf]

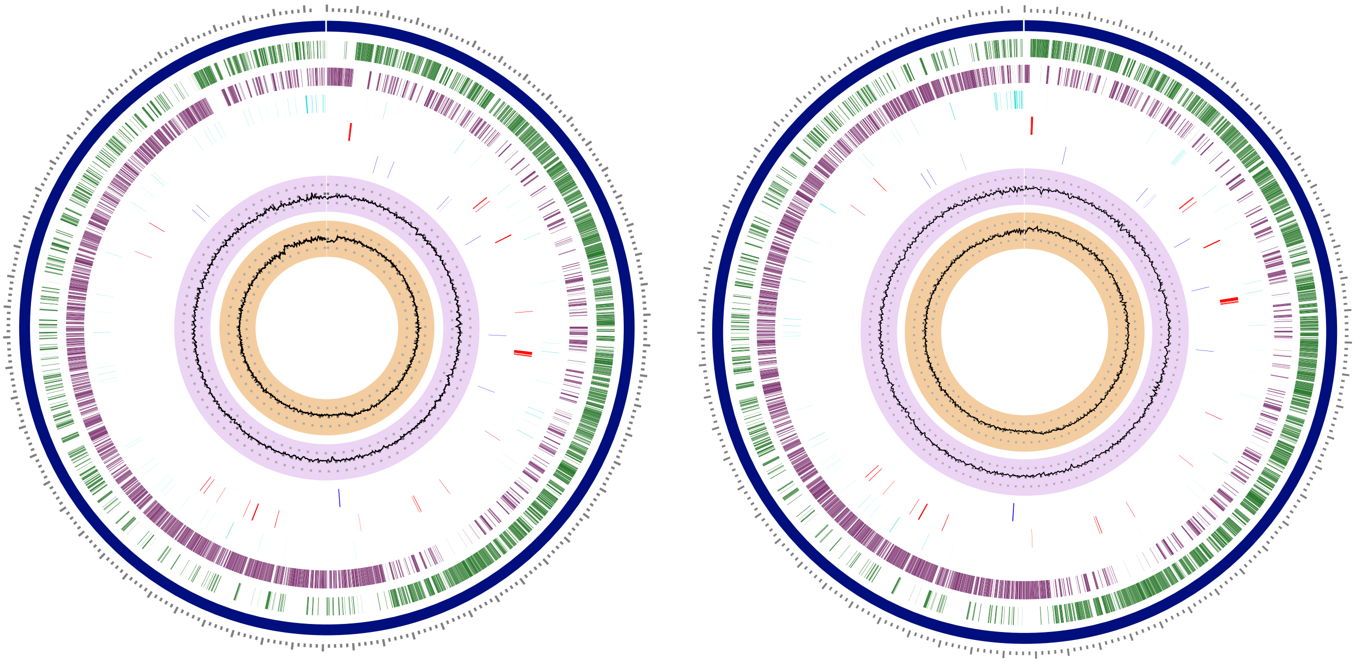

(a) *L. plantarum* DKL3

(b) *L. plantarum* JGR2

Figure 1S: **Circular representation of the assembled genomes of *L. plantarum* DKL3 and JGR2** drawn using PATRIC (v 3.6.12, https://www.patricbrc.org/)**.** From outer to inner rings, contigs ordered as per WCFS1 chromosome, CDS on the forward strand, CDS on the reverse strand, RNA genes, CDS with homology to known antimicrobial resistance genes, transport-related genes, GC content, and GC skew.
