# Supplementary material for: Isolation, characterization and comparative genomics of potentially probiotic Lactiplantibacillus plantarum strains from Indian foods
Source: Sci Rep. 2022 Feb 4;12:1940. doi: 10.1038/s41598-022-05850-3 (PMC8816928; doi:10.1038/s41598-022-05850-3)
Supplement: Supplementary file 3 — Supplementary Figure S2. [file 41598_2022_5850_MOESM3_ESM.pdf]

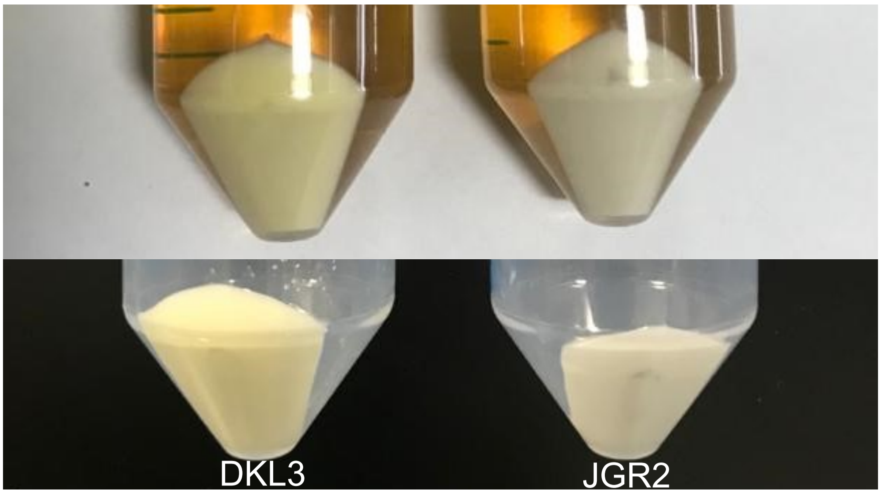

Figure 2S: **Color variation in the biomass of *L. plantarum* DKL3 and JGR2** depicting yellowish cell pellet of DKL3 as compared to that of JGR2.
