# Supplementary material for: Isolation, characterization and comparative genomics of potentially probiotic Lactiplantibacillus plantarum strains from Indian foods
Source: Sci Rep. 2022 Feb 4;12:1940. doi: 10.1038/s41598-022-05850-3 (PMC8816928; doi:10.1038/s41598-022-05850-3)
Supplement: Supplementary file 4 — Supplementary Figure S3. [file 41598_2022_5850_MOESM4_ESM.pdf]

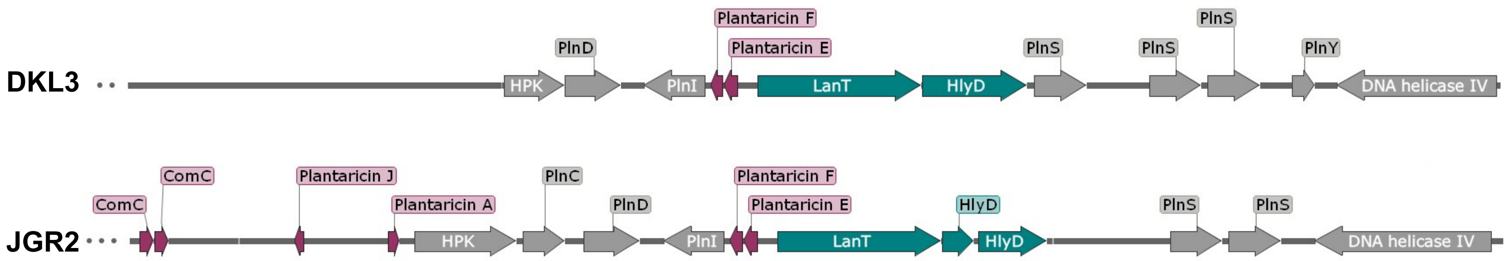

Figure 3S: **Bacteriocin-related genes identified in *L. plantarum* DKL3 and JGR2 using BAGEL4 (http://bagel4.molgenrug.nl/).** Core genes are indicated in maroon, transport-related genes in green and other genes in grey. The arrows indicate the directions of the ORFs.
